# New Fluoride-arsenide Diluted Magnetic Semiconductor (Ba,K)F(Zn,Mn)As with Independent Spin and Charge Doping

**DOI:** 10.1038/srep36578

**Published:** 2016-11-22

**Authors:** Bijuan Chen, Zheng Deng, Wenmin Li, Moran Gao, Qingqing Liu, C. Z. Gu, F. X. Hu, B. G. Shen, Benjamin Frandsen, Sky Cheung, Liu Lian, Yasutomo J. Uemura, Cui Ding, Shengli Guo, Fanlong Ning, Timothy J. S. Munsie, Murray Neff Wilson, Yipeng Cai, Graeme Luke, Zurab Guguchia, Shingo Yonezawa, Zhi Li, Changqing Jin

**Affiliations:** 1 Beijing National Laboratory for Condensed Matter Physics, Institute of Physics, Chinese Academy of Sciences, Beijing 100190, China; 2 Department of Physics, Columbia University, New York, New York 10027, USA; 3 Department of Physics, Zhejiang University, Hangzhou 310027, China; 4 Department of Physics and Astronomy, McMaster University, Hamilton L8S 4M1, Canada; 5 Physik Institut der Universität Zürich, Winterthurerstrasse 190, CH-8057 Zürich, Switzerland; 6 Department of Physics, Graduate School of Science, Kyoto University, Kyoto 606-8502, Japan; 7 School of Materials Science and Engineering, Hefei University of Technology, Hefei, 230009, China; 8 Collaborative Innovation Center of Quantum Matter, Beijing 100190, China

## Abstract

We report the discovery of a new fluoride-arsenide bulk diluted magnetic semiconductor (Ba,K)F(Zn,Mn)As with the tetragonal ZrCuSiAs-type structure which is identical to that of the “1111” iron-based superconductors. The joint hole doping via (Ba,K) substitution & spin doping via (Zn,Mn) substitution results in ferromagnetic order with Curie temperature up to 30 K and demonstrates that the ferromagnetic interactions between the localized spins are mediated by the carriers. Muon spin relaxation measurements confirm the intrinsic nature of the long range magnetic order in the entire volume in the ferromagnetic phase. This is the first time that a diluted magnetic semiconductor with decoupled spin and charge doping is achieved in a fluoride compound. Comparing to the isostructure oxide counterpart of LaOZnSb, the fluoride DMS (Ba,K)F(Zn,Mn)As shows much improved semiconductive behavior that would be benefit for further application developments.

Diluted magnetic semiconductors (DMSs) are semiconductors where a small part of the component ions are substituted by magnetic ions, leading to a variety of cooperative effects and exhibiting outstanding properties and functionalities^1^,^2^,^3^,^4^,^5^,^6^. For example, a carrier-mediated magnetism in a DMS can be tuned by the carrier density through an applied electric field^2^,^3^. The discovery of Mn-doped III-V ferromagnetic semiconductors, such as (In,Mn)As^4^, (Ga,Mn)N^5^ and (Ga,Mn)As^6^, has triggered extensive research with the intention of exploring new DMSs and enables examination of collective magnetic phenomena in a well-controlled environment^7^,^8^,^9^,^10^. At the same time, applications in sensors and memory devices as well as for computing with electronic spins can be envisaged^2^,^11^,^12^,^13^.

However, the typical systems based on III-V semiconductors face serious challenges. In (Ga,Mn)As, for example, substitution of divalent Mn^2+^ into trivalent Ga^3+^ sites has severely limited chemical solubility, resulting in metastable specimens only available as epitaxial thin films, which has led to considerable controversy about mechanisms of ferromagnetism in (Ga,Mn)As. Therefore, seeking decoupled of spin and charge injections with bulk DMSs become one of major missions for DMS materials^14^. Recently, a new type of DMS Li(Zn,Mn)As was reported to be a bulk DMS material with a ferromagnetic Curie temperature (*T*_*C*_) up to 50 K^15^. In this material, spins are doped via isovalent (Zn^2+^, Mn^2+^) substitution, while charges are provided by off-stoichiometry of the Li concentrations. Shortly after, another new type of bulk ferromagnetic DMS (Ba,K)(Zn,Mn)_2_As_2_ was synthesized with much higher *T*_*C*_ up to 230 K^16^,^17^. More recently, several types of bulk DMSs with decoupled spin & charge doping have been successfully fabricated, including Li(Zn,Mn)P^18^, (La,Ca)(Zn,Mn)SbO^19^, (La,Ba)(Zn,Mn)AsO^20^, (La,Sr)(Cu,Mn)SO^21^, (Sr,Na)(Cd,Mn)_2_As_2_^22^, and (*A*,Na)(Zn,Mn)_2_As_2_ (*A* = Ca, Sr,)^23^,^24^.

The successful discovery of superconductivity in the F doped LaFeAsO has aroused extensively studies on the compounds with the ZrCuSiAs-type structure and the relationship with other structures^19^,^20^,^25^. In this article, we report a new fluoride-arsenide ferromagnetic semiconductor (Ba,K)F(Zn,Mn)As, which shares the same structure as that of “1111” iron-based superconductor Sr_1−x_Sm_x_FFeAs^25^ and Ba_1−x_Sm_x_FFeAs^26^ (the tetragonal ZrCuSiAs-type structure). The compound of (Ba,K)F(Zn,Mn)As is isostructural to its variants, *i.e.*, antiferromagnets and superconductors with lattice matching within 5%^26^,^27^, which could provide the possibility to make junction devices with these materials. Compared to BaZn_2_As_2_^28^ (the parent compound of the high *T*_*C*_ DMS (Ba,K)(Zn,Mn)_2_As_2_) and the isostructure oxide counterpart of LaOZnSb^29^, the fluoride-based parent compound BaFZnAs is more ionic hence semiconductive. Via (Ba,K) substitution to dope hole carriers and (Zn,Mn) substitution to supply local spins, this system reaches decoupled spin & charge doping exhibiting ferromagnetic order with *T*_*C*_ up to 30 K and related negative magnetoresistance. Compared to the oxides compounds, fluoride compounds are able to form mostly ionic bonds, resulting in fluorines’s high electronegativity^30^. Hence, DMS achieved in fluoride compound seem to provide materials with several useful properties and promote the development of new materials for modern applications.

## Results and Discussion

### Crystal Structure of (Ba_1**−** *x*_K_*x*_)F(Zn_1−*y*_Mn_*y*_)As

**Figure 1:**
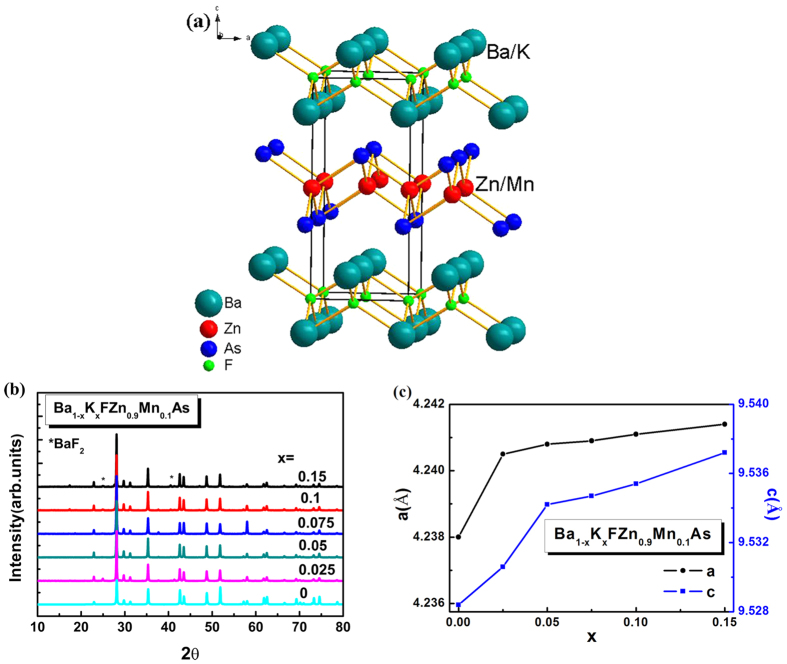
(**a**) Tetragonal ZrCuSiAs-type crystal structure of (Ba,K)F(Zn,Mn)As. (**b**) Powder XRD patterns of (Ba_1−*x*_K_*x*_)F(Zn_0.9_Mn_0.1_)As taken at room temperature. Traces (*) represent the impurity phase of BaF_2_. (**c**) Lattice constants of *a* axis and *c* axis of (Ba_1−*x*_K_*x*_)F(Zn_0.9_Mn_0.1_)As obtained from XRD.

The structure of (Ba,K)F(Zn,Mn)As, as shown in Fig. 1a, is the same as that of the parent compound BaFZnAs^31^. It is found that the majority of peaks in the XRD pattern can be well indexed to main phase, except for some tiny peaks from the nonmagnetic impurity phase of BaF_2_. The unit cell of BaFZnAs is composed of two different layers: ZnAs-layer with [ZnAs_4_] tetrahedron and BaF-layer with [BaF_4_] tetrahedron. The two different layers are stacked in an AB AB sequence along the *c*-axis, resulting in the compound’s quasi two-dimensional nature. The lattice parameters were calculated to be *a* = 4.2380 Å and *c* = 9.5284 Å, which are similar with that of LaOZnSb (*a* = 4.2267 Å and *c* = 9.538 Å), the parent compound for “1111” oxide-arsenide diluted magnetic semiconductors^19^. This is caused by big size difference between La^3+^ (1.061 Å) and Ba^2+^ (1.35 Å) as well as between Sb^3−^ (0.76 Å) and As^3−^ (0.58 Å). Figure 1b shows the X-ray diffraction patterns of (Ba_1−*x*_K_*x*_)F(Zn_0.9_Mn_0.1_)As for *x* = 0, 0.025, 0.05, 0.075, 0.1 and 0.15, respectively. The lattice parameters monotonically increase with the increase of K doping, as shown in Fig. 1c, due to the slightly larger ionic radius of K^+^ (1.38 Å) compared with that of Ba^2+^ (1.35 Å). However, the deviation from the linear relation is likely from the incorporation of defects such as antisites, interstitials etc that are common in these type of compounds. These results indicate the successful chemical doping of K.

### Magnetic Properties of (Ba_1−*x*_K_*x*_)F(Zn_1−*y*_Mn_*y*_)As

**Figure 2:**
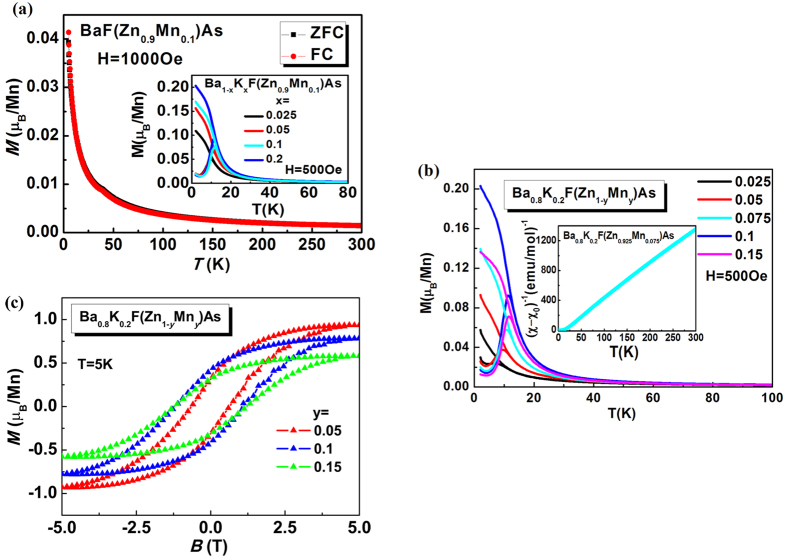
(**a**) DC *M*(*T*) measurements measured in *H* = 1000 G for BaF(Zn_0.9_Mn_0.1_)As without charge doping. Inset shows the magnetization curve of (Ba_1−*x*_K_*x*_)F(Zn_0.9_Mn_0.1_)As specimens with several charge doping. (**b**) *M*(*T*) measured in *H* = 500 G for (Ba_0.8_K_0.2_)F(Zn_1−*y*_Mn_*y*_)As with ZFC and FC procedures. Inset shows the temperature dependence of the inverse susceptibility for (Ba_0.8_K_0.2_)F(Zn_0.925_Mn_0.075_)As. (**c**) *M*(*H*) curves of (Ba_0.8_K_0.2_)F(Zn_1−*y*_Mn_*y*_)As samples at temperature *T* = 5 K.

In DMSs, competing interactions between ferromagnetic and anti-ferromagnetic along with the spin-exchange interactions between local magnetic moments and carriers in magnetic systems could contribute to a variety of magnetic structures and critical phenomena^12^. Particularly, the long-range magnetic order nature of the magnetic interactions mediated by the conduction carriers leads to a diversity of cooperative effects. Figure 2a shows the temperature dependence of the magnetization *M*(*T*) in zero-field-cooling (ZFC) and field-cooling (FC) procedures under *H* = 1000 G for BaF(Zn_0.9_Mn_0.1_)As. No ferromagnetic order is observed when only doping Mn. BaF(Zn_0.9_Mn_0.1_)As is paramagnetic from room temperature down to 2 K. Inset of Fig. 2a shows the magnetization curve of (Ba_1−*x*_K_*x*_)F(Zn_0.9_Mn_0.1_)As specimens with *x* = 0.025, 0.05, 0.1 and 0.2, respectively. Signatures of ferromagnetic order are seen in the curves at temperatures of 20 K *~*30 K. *T*_*C*_ monotonously increases with the increase of K doping. These results indicate that only joint carrier doping via (Ba,K) substitution with spin doping arising from (Zn,Mn) substitution can give rise to the ferromagnetic order. Hole doping drives the system toward ferromagnetism^32^,^33^,^34^,^35^. Meanwhile, the ferromagnetic order is tuned by carrier concentration *x* and the spin level *y*, which is consistent with the carrier-mediated mechanism of the ferromagnetism as described by the Rudermann-Kittel-Kasuya-Yosida (RKKY) model or the Zener model^12^,^36^. Figure 2b shows the *M*(*T*) in ZFC and FC procedures under *H* = 500 G for the (Ba_0.8_K_0.2_)F(Zn_1−*y*_Mn_*y*_)As samples with *y* = 0.025, 0.05, 0.075, 0.1 and 0.15, respectively. The maximum *T*_*C*_ is 30 K for optimal Mn doping (*y* = 0.1). Further Mn doping results in *T*_*C*_gradually decreasing, as shown in Fig. 2b. Above *T*_*C*_, the susceptibility *χ* can be fit to Curie-Weiss formula,


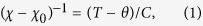


where *χ*_0_ is a temperature-independent paramagnetic term, *C* is the Curie constant, and *θ* is the Weiss temperature. The positive value of *θ* found for (Ba_0.8_K_0.2_)F(Zn_0.925_Mn_0.075_)As [inset of Fig. 2(b)] indicates a ferromagnetic interaction between Mn^2+^ ions.

Figure 2c shows the field dependence of magnetization *M*(*H*) curves of (Ba_0.8_K_0.2_)F(Zn_1−*y*_Mn_*y*_)As with *y* = 0.05, 0.1 and 0.15 at *T* = 5 K with corrections by subtracting the small *H*-linear component presumably from remaining paramagnetic spins and/or field-induced polarization^17^. For *x* fixed to 20%, the maximum saturation magnetization (*M*_*sat*_) is 0.9 μ_B_ when *y* = 5%. However, upon further increasing Mn concentration, *M*_*sat*_ of the samples decreases to 0.6 μ_B_ with the increasing Mn doping. These decreasing trends of *M*_*sat*_ probably reflect the competition between the short-range antiferromagnetic superexchange of nearest-neighbor Mn moments and a longer-range ferromagnetic interaction of distant Mn moments regulated by hole carriers^32^, *i.e.* the RKKY-like interaction. The direct antiferromagnetic coupling between the Mn-Mn pairs causes G-type antiferromagnetic order in BaFMnAs at *T*_*N*_ = 338 K^27^. A similar trend was found in other magnetic ion doped systems^15^,^18^,^23^,^37^.

### Muon spin relaxation (μSR) measurements

**Figure 3:**
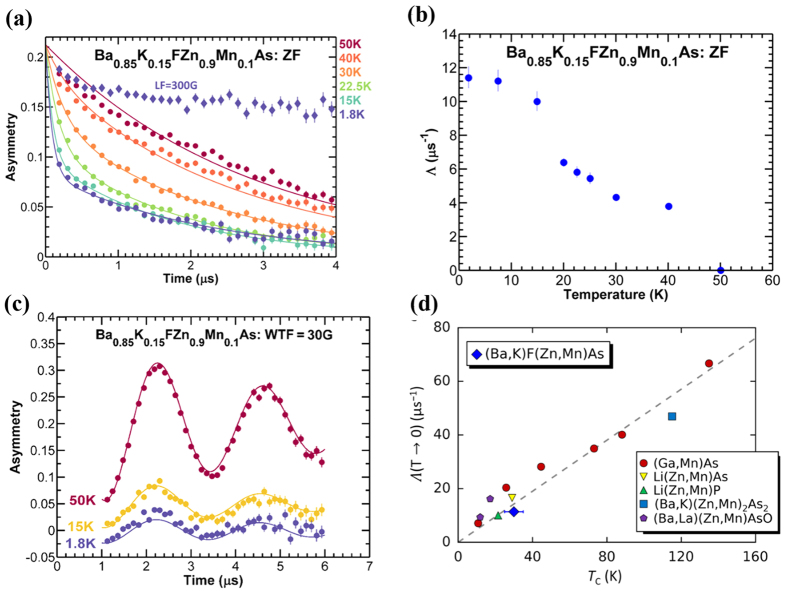
Results of *μ*SR measurements of (Ba_0.85_K_0.15_)F(Zn_0.9_Mn_0.1_)As. (**a**) Zero-field μSR spectra at various temperatures. The colored points represent the experimental data, and the solid curves represent the fits described in the text. A longitudinal-field measurement taken at 2 K is shown by the blue diamonds. (**b**) Fast relaxation rate *Λ* obtained from fits described in the text. The error bars represent the estimated standard deviations of the refined parameters. (**c**) Weak-transverse-field measurements at various temperatures, offset vertically for clarity. (**d**) Low-temperature relaxation rate *Λ* plotted against the ferromagnetic ordering temperature *T*_C_ for various DMS systems, including the present work, exhibiting a linear relationship with a common slope. The gray dashed line is the best linear fit.

The ability of *μ*SR to determine the temperature dependence of the magnetically ordered volume fraction and the magnetic order parameter has made it a valuable tool for studying many other DMS materials^15^,^17^,^20^,^23^,^24^,^38^,^39^,^40^,^41^,^42^. The availability of bulk specimens allowed us to perform conventional *μ*SR on (Ba_1−*x*_K_*x*_)F(Zn_1−*y*_Mn_*y*_)As. To further probe the magnetic order in this system, we performed *μ*SR measurements on a sample of (Ba_0.85_K_0.15_)F(Zn_0.9_Mn_0.1_)As. Zero-field (ZF) *μ*SR spectra taken at various temperatures are displayed in Fig. 3a. The development of rapid relaxation indicates the presence of magnetic order starting around 40 K and below. When a longitudinal field (LF) of 300 G is applied parallel to the initial muon spin direction, nearly the full asymmetry is recovered, confirming that the relaxation in ZF is due primarily to static magnetic order rather than dynamically fluctuating magnetic moments. The LF spectrum taken at 2 K is shown as blue diamonds in Fig. 3a.

The ZF spectra can be well fit by a sum of two exponential functions, a “fast” component whose rate is proportional to the magnetic order parameter, and a “slow” component capturing the relaxation from the paramagnetic regions of the sample and the “1/3 tail” of the ordered regions. The lack of coherent oscillations in the ZF spectra reflect the spatial disorder of the magnetic moments due to the random distribution of the magnetic dopants, and has also been observed in other DMS systems^23^. The temperature dependence of the fast relaxation rate *Λ* is displayed in Fig. 3b, exhibiting a monotonic increase as the temperature is lowered below 40 K and reaching a maximum value of 11.5+/− 0.7 μs^−1^ at the lowest measurement temperature (2 K). From these results, we assign the onset transition temperature (*T*_*C*_°) to be around 40 K ~ 50 K, but slightly higher than *T*_*C*_ = 30 K determined from magnetization measurements. This suggests a rather broad transition, with the higher *μ*SR onset temperature corresponding to a partial volume fraction that orders first, while the lower *T*_*C*_ from bulk magnetization reflects the point where the majority of the sample becomes ordered.

To verify that the full sample volume orders magnetically at low temperature, we performed weak-transverse field (WTF) *μ*SR measurements. Representative WTF spectra are shown in Fig. 3c, offset vertically for clarity. The oscillating amplitude, which is proportional to the non-magnetically ordered volume fraction, is gradually reduced as the temperature is lowered, again pointing to a broad magnetic transition and confirming the presence of magnetic order in a large majority of the sample volume. From the ratio of the oscillating amplitude at 2 K and 50 K, we estimate the magnetically ordered volume fraction to be 0.85+/− 0.1 at 2 K. Background contributions and minor phase impurities may contribute to the non-magnetically-ordered volume fraction, but these results nevertheless confirm the intrinsic nature of the magnetic order in this material.

The magnetically ordered volume fraction reaches 50% at approximately 30 K, coinciding with the transition temperature determined by magnetization. Broadly assigning the transition temperature to be *T*_*C*_ = 30 K+/− 5 K, the low-temperature ZF relaxation rate *Λ (T* → 0) = 11.5+/− 0.7 μs^−1^ can be plotted against *T*_C_, as shown in Fig. 3d. We compare the present result on (Ba_0.85_K_0.15_)F(Zn_0.9_Mn_0.1_)As with the earlier DMS systems of the (Ga,Mn)As^41^ system, Li(Zn,Mn)As^15^ system, (Ba,K)(Zn,Mn)_2_As_2_^17^ system, and (La,Ba)(Zn,Mn)AsO^20^ system in a plot of the low temperature relaxation rate *Λ* and the Curie temperature *T*_*C*_. This system lies quite close to the roughly linear relationship between *Λ (T* → 0) and *T*_C_ exhibited by the other systems, which suggests that exchange interaction supporting ferromagnetic coupling in these systems has a common mechanism for the ferromagnetism in all of these materials.

### Bandgap and Electronic Structure of parent compound BaFZnAs

**Figure 4:**
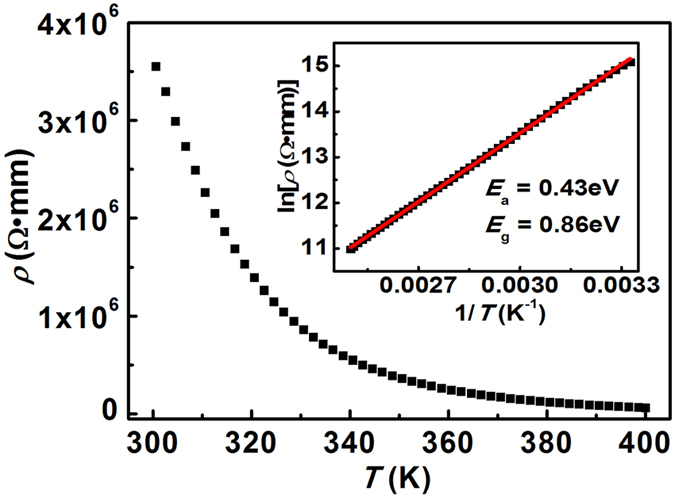
Temperature dependence of resistivity for parent compound BaFZnAs sample. Inset displays the resistivity of BaFZnAs in the ln*ρ* vs. 1/*T* plot. The red curve is a fit to *ρ*(*T*) = *ρ*_*0*_ exp(*E*_*a*_/*k*_*B*_*T*).

High quality polycrystalline BaFZnAs was reported to be a semiconductor in our previous work^31^. Figure 4 shows the temperature dependence of resistivity of BaFZnAs, indicating a semiconductor behavior. Inset shows the ln*ρ* vs. 1/*T* plot of BaFZnAs. The red curve is the fit by the formula,


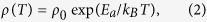


where *ρ*_*0*_ is the preexponential constant, *k*_*B*_ is the Boltzmann constant, and *E*_*a*_ is the activation energy, at high-temperature region (300 to and 400 K). The high-*T* linear region should be attributed to the intrinsic region of *ρ*. The fit gave an intrinsic *E*_*a*_ value of 0.43 eV, *i.e.* the bandgap *E*_*g*_ = 2*E*_*a*_ = 0.86 eV, which is slightly larger than that of BaFMnAs (0.73 eV)^27^. The band gap of BaFeAsZn is much larger than that of BaZn_2_As_2_ (0.23 eV)^28^ and the isostructure oxide counterpart of LaOZnSb^29^, suggesting the larger ionicity of fluoride counterpart than that of BaZn_2_As_2_ and LaOZnSb.

**Figure 5:**
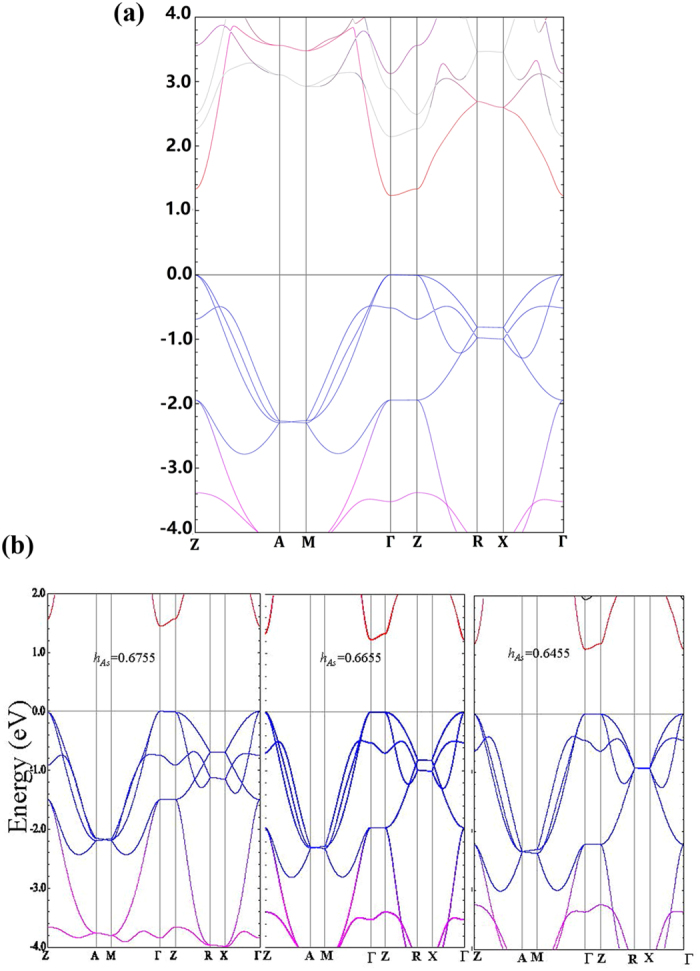
(**a**) Band structure of BaFZnAs: the weight of As (Zn) *p (s*)-orbitals is in blue (red). (**b**) Band gaps with different heights of As for *h*_As_ = 0.6755, *h*_As_ = 0.6655 and *h*_As_ = 0.6455, respectively, which reveals that the band gap of BaFZnAs will increase (decrease) if the As is slightly moved to the BaF-(Zn) layer.

To understand the electronic structure of the host semiconductor BaFZnAs, we also calculated the bandgap of BaFZnAs by first-principles calculations. The underestimated value of bandgap from GGA results happened in LaOZnAs^28^, but a reasonable result was obtained with modified Becke-Johnson local density approximation (MBJLAD). Here we apply the same approach, *i.e.* the MBJLAD to estimate the band gap of BaFZnAs. The calculation gives rise to a gap of 1.2 eV, as shown in Fig. 5a, which is a little larger than the value from transport experiments. Taking the improved conductivity caused by crystal defects into consideration, the bandgap (0.86 eV) of BaFZnAs from transport may be smaller than that of real value. We conclude that the value estimated from MBJLDA calculation provide a reasonable bandgap for BaFZnAs.

In Fig. 5a, both As 4*p* (in blue) and Zn 4*s* states (in red) are located around the Fermi level (−2.0 to 2.0 eV) in BaFZnAs. The topmost valence bands and the lowest conducting band are dominated by the As 4*p*-orbitals and the Zn 4*s*-orbitals, respectively. The bandwidth of Zn 4*s* is determined by two kinds of 4*s*-4*s* hybridization, *i.e.*, direct 4*s*-4*s* hybridization in the Zn plane and indirect 4*s*-4*s* hybridization mediated by the As 4*p*-orbitals. The indirect 4*s*-4*s* hybridization mediated by As 4*p*-orbitals is proportional to


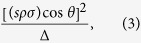


where (*sρσ*) is the two-center integral of Zn 4*s* and A*s* 4*p*-orbitals, *θ* is the As-Zn-Zn angle in yz/xz plane, and Δ is single particle energy difference of Zn 4*s* and As 4*p*-orbitals. Our calculated band gaps with different heights reveal that the band gap of BaFZnAs will increase (decrease) if the As is slightly moved to the BaF- (Zn) layer, as clearly shown in Fig. 5b. The As height dependent evolution of band gap can be interpreted by the competition of direct and indirect 4*s*-4*s* hybridization. The indirect 4*s*-4*s* hybridization will enhanced when the As is moved to the Zn layer, and the bandwidth of Zn 4*s* will increase leading to smaller band gap. The band gap will be dominated by the direct 4*s*-4*s* hybridization if the As is moved to the BaF-layer, leading to larger band gap. The delicate band gap of BaFZnAs and its analogies is determined by the completion of direct 4*s*-4*s* hybridization and indirect hybridization mediated by As 4*p*-orbitasl. The absence of dispersion along *R*-*X* line also suggests quasi two dimensional character of the band structure in BaFZnAs (Fig. 5a).

### Transport Properties of (Ba_1**−** *x*_K_*x*_)F(Zn_1−*y*_Mn_*y*_)As

**Figure 6:**
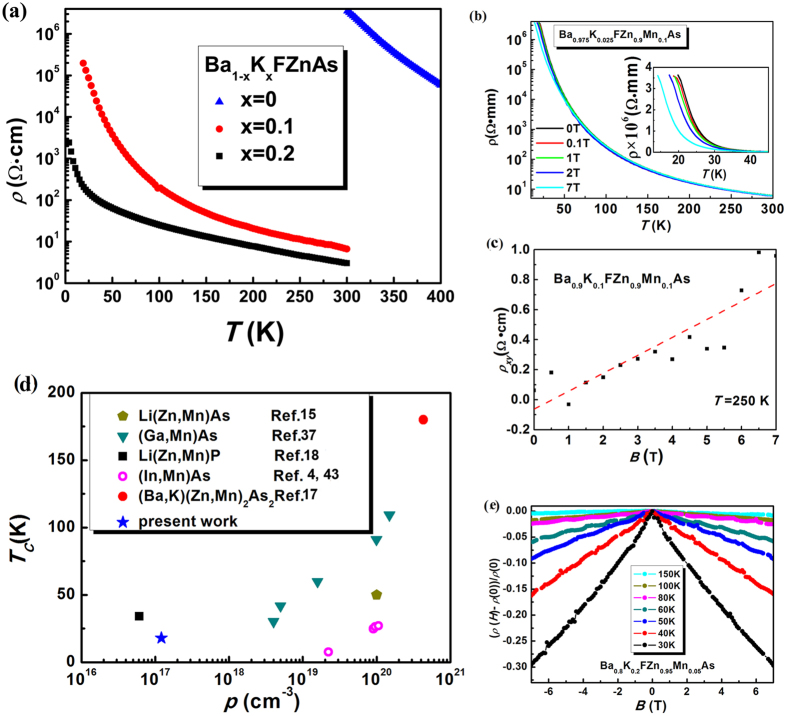
(**a**) Resistivity of Ba_1−*x*_K_*x*_FZnAs for *x* = 0, 0.1, 0.2 samples. (**b**) *ρ*(*T*) of (Ba_0.925_K_0.075_)F(Zn_0.9_Mn_0.1_)As under various fields. Inset shows the enlarged *ρ*(*T*) curve for (Ba_0.925_K_0.075_)F(Zn_0.9_Mn_0.1_)As under various fields at low temperatures. (**c**) Hall effect measurements of (Ba_0.1_K_0.1_)F(Zn_0.9_Mn_0.1_)As specimen at *T* = 250 K. (**d**) Correlation between *T*_*C*_ and the hole concentration for various DMS systems. The blue stars represent the present work. (**e**) Negative magnetoresistance of (Ba_0.8_K_0.2_)F(Zn_0.95_Mn_0.05_)As at different temperatures, which can be defined as [*ρ*(*H*) − *ρ*(0)]/*ρ*(0).

The temperature dependence of electrical resistivity *ρ*(*T*) of (Ba_1−*x*_K_*x*_)FZnAs for *x* = 0, 0.1, and 0.2 is shown in Fig. 6a. All the specimens exhibit typical semiconducting behavior over the entire temperature range. For the *x* = 0 specimen, *ρ*(*T*) is on the order of 10^6^ Ω cm at room temperature, much larger than that of BaZn_2_As_2_, another parent compound of diluted magnetic semiconductor^17^. Doping K atoms into Ba sites introduces hole carriers, leading to the much smaller resistivity in (Ba,K)FZnAs than that of BaFZnAs. Figure 6b shows the resistivity *ρ*(*T*) for (Ba_0.925_K_0.075_)F(Zn_0.9_Mn_0.1_)As under various magnetic field. The *ρ*_*H*_(*T*) diverges from each other under different fields and increases drastically below *T*_*C*_. Resistivity was beyond our measurement limitation below 20 K that is primarily caused by the more ionic nature of fluorides in sharp contrast to low resistance oxide counterparts wherein more covalence chemical bonding is expected^20^. The extremely large resistivity precluded Hall effect measurements on the polycrystalline specimens at low temperature. However, the Hall effect can be measured at high temperature (*T* = 250 K), as shown in Fig. 6c. The positive Hall coefficient demonstrates dominated hole type carriers by 10% K-substitution in the system. In a single-band model, the Hall coefficient R_*H*_ is associated with carrier density (*p*) as R_*H*_ = 1/*p*e. Therefore, we calculate the hole concentration to be 1.21 × 10^17^ cm^−3^. The Zener model description of ferromagnetism in Ga_1−*x*_Mn_*x*_As reveals that the higher values of *T*_*C*_ are predicted for materials containing larger concentrations of holes and magnetic ions^12^, which suggests that a higher *T*_*C*_ could be achieved in the present system if further charging doping could be accompanied by a corresponding increase in hole concentrations^32^. Figure 6d compares the hole concentrations and ferromagnetic transition temperatures of (Ba,K)F(Zn,Mn)As) to those of other DMS systems. The hole concentration of (Ba_0.8_K_0.2_)F(Zn_0.95_Mn_0.05_)As is comparable with that of Li(Zn,Mn)P system, while is more than two order of magnitude smaller than that of typical metallic DMS ferromagnets^15^,^17^,^23^,^43^. It is interesting to noticed that the present work and Li(Zn,Mn)P^18^ system both exhibit ferromagnetism with relatively high *T*_*C*_ while the carriers still remain semiconducting. The relationship between *T*_*C*_ and hole concentration exhibited by (Ga,Mn)As^37^, (In,Mn)As^4^,^43^, Li(Zn,Mn)As^15^ and (Ba,K)(Zn,Mn)_2_As_2_^17^ systems suggests that further charge and spin doping would cause (Ba,K)F(Zn,Mn)As to be metallic and achieve magnetically order at a higher *T*_*C*_^3^,^9^,^12^,^15^,^17^,^18^,^44^. The parallel expectations are valid to the present fluoride DMS.

There are many factors in magnetic semiconductors that can produce a sizable magnetoresistance. Under many conditions, the reduction of spin-dependent scattering by aligning the spins in an applied field leads to negative magnetoresistance^2^,^37^. The resistivity dependence of magnetic field *ρ*(*H*) for (Ba_0.8_K_0.2_)F(Zn_0.95_Mn_0.05_)As at several temperatures is shown in Fig. 6e. Negative magnetoresistance is observed in the whole temperature range of (Ba_0.8_K_0.2_)F(Zn_0.95_Mn_0.05_)As. The negative magnetoresistance is far from saturation even in rather high magnetic field, at which spin orientation is fully aligned. In this condition, an orbital effect resulting from the destructive influence of the magnetic field on the interference of scattered waves prevents the negative magnetoresistance from saturating even in low temperatures and rather strong magnetic fields.

## Conclusion

We presented the successful synthesis of a new fluoride-arsenide ferromagnetic DMS (Ba,K)F(Zn,Mn)As via decoupled charge and spin doping. It is the first time that ferromagnetic ordering has been observed in a fluoride-arsenide semiconductor with Mn doping. The ferromagnetism of this system is mediated with decoupled spin & charge doping. The magnetization showed bulk ferromagnetism with *T*_*C*_ around 15 K ~30 K for various composition of (Ba,K)F(Zn,Mn)As. The *μ*SR measurements confirmed the intrinsic nature of the long range magnetic order in the entire volume at low temperature. Compared to parent compounds of (Ba,K)(Zn,Mn)_2_As_2_ and (La,Ca)O(Zn,Mn)Sb, *i.e.*, BaZn_2_As_2_ and LaOZnSb, the fluoride-based parent compound BaFZnAs is more ionic and semiconductive, which would be benefit for further application developments.

Our results suggest that further studies of transition-metal-based ZrCuSiAs type structure of DMS materials in general, and other members of the fluoride-arsenide family in particular, are warranted.

## Methods

### Synthesis of Polycrystalline Samples

Polycrystalline (Ba,K)F(Zn,Mn)As specimens were synthesized via conventional solid-state reactions, a procedure similar to that employed for (Sr,Na)(Zn,Mn)_2_As_2_^23^. Firstly, potassium arsenide and barium arsenide precursors were synthesized from stoichiometric mixtures of potassium pieces, arsenide powders and barium pieces in evacuated silica-glass ampules at 500 °C and 700 °C for 20 h, respectively. Secondly, powders of potassium arsenide, barium arsenide, barium fluoride, and high-purity zinc and manganese were mixed and ground in stoichiometric quantities, and pressed into pellets. The pellets were loaded into tantalum tubes filled with high-purity Ar gas, and then were sealed in evacuated quartz tubes. The mixtures were sintered at 750 °C for 20 h before they were slowly decreased to room temperature. All the synthesis processes were carried out in high-purity Ar atmosphere (O_2_ < 0.1 ppm, H_2_O < 0.01 ppm).

### Structural, Magnetic and Electronic Measurements

The phase purity of the resulting powers was examined by powder X-ray diffraction (XRD; Philips X’pert diffractometer) using Cu-K_α_ radiation at room temperature. The crystal structure and lattice constants were calculated by Rietveld refinement using the GSAS software package. The DC magnetic susceptibility measurements were performed with a superconducting quantum interference device (SQUID-VSM; Quantum Design). The electronic transport measurements were measured by the four-probe technique using silver paste electrodes on a Quantum Design PPMS. Muon spin relaxation (*μ*SR) measurements were performed at TRIUMF in Vancouver, Canada.

### Band Structure Calculations

The first-principle electronic structure calculations were performed using experimental crystallographic parameters^31^ and the full-potential linearized augmented plane wave (LAPW) method implemented in the WIEN2k package^45^. The general gradient approximation (GGA)^46^ was used for the exchange-correlation potential. However, we found it underestimated the bandgaps of BaFZnAs. We then examined with modified Becke-Johnson local density approximation (MBJLAD) provided better results. The LAPW sphere radius was set to 2.40, 1.00, 2.49 and 2.37 Bohr for Ba, F, Zn and As, respectively. The energy cut-off was set to *R*_*min*_*K*_*max*_ = 8.0 and the *k*-point sample was set to 40 × 40 × 18.

## Additional Information

**How to cite this article**: Chen, B. *et al*. New Fluoride-arsenide Diluted Magnetic Semiconductor (Ba,K)F(Zn,Mn)As with Independent Spin and Charge Doping. *Sci. Rep.* **6**, 36578; doi: 10.1038/srep36578 (2016).

**Publisher's note**: Springer Nature remains neutral with regard to jurisdictional claims in published maps and institutional affiliations.
